# The Influence of Age and Skull Conductivity on Surface and Subdermal Bipolar EEG Leads

**DOI:** 10.1155/2010/397272

**Published:** 2010-01-10

**Authors:** Katrina Wendel, Juho Väisänen, Gunnar Seemann, Jari Hyttinen, Jaakko Malmivuo

**Affiliations:** ^1^Department of Biomedical Engineering, Tampere University of Technology, Korkeakoulunkatu 3, P.O. Box 692, 33101 Tampere, Finland; ^2^Institute of Biomedical Engineering, Karlsruhe Institute of Technology, 76131 Karlsruhe, Germany

## Abstract

Bioelectric source measurements are influenced by the measurement location as well as the conductive properties of the tissues. Volume conductor effects such as the poorly conducting bones or the moderately conducting skin are known to affect the measurement precision and accuracy of the surface electroencephalography (EEG) measurements. This paper investigates the influence of age via skull conductivity upon surface and subdermal bipolar EEG measurement sensitivity conducted on two realistic head models from the Visible Human Project. Subdermal electrodes (a.k.a. subcutaneous electrodes) are implanted on the skull beneath the skin, fat, and muscles. We studied the effect of age upon these two electrode types according to the scalp-to-skull conductivity ratios of 5, 8, 15, and 30 : 1. The effects on the measurement sensitivity were studied by means of the half-sensitivity volume (HSV) and the region of interest sensitivity ratio (ROISR). The results indicate that the subdermal implantation notably enhances the precision and accuracy of EEG measurements by a factor of eight compared to the scalp surface measurements. In summary, the evidence indicates that both surface and subdermal EEG measurements benefit better recordings in terms of precision and accuracy on younger patients.

## 1. Introduction

 Clinical electroencephalography (EEG) and evoked potential (EP) recordings such as the visually evoked potentials (VEPs) demand high signal-to-noise ratios (SNRs), minimization of skin artifacts, and high accuracy, to name a few important criteria. Subdermal needle electrodes (a.k.a. subcutaneous needle electrodes) are commonly used in clinical electromyography (EMG), which are inserted into the muscles of interest. It is less commonly known that these subdermal needle electrodes also record continuous EEGs and EPs in intensive care units (ICU) [1–4]. The measurement setup is achieved by inserting the needle nearly tangentially to the skin so that it is stabilized and the recording tip touches the skull. Furthermore, these recordings offer higher SNRs with lower proclivity of standard measurement artifacts when compared with traditional surface measurements and are more suitable for long-term EEG monitoring in the ICU. Higher SNR requires less averaging, thus yielding faster and more accurate diagnostic measurements. We believe that clinical EEGs and EPs such as the VEP could adopt the subdermal measurement setup, thus placing the lead on the skull bypassing the artifact-prone skin. 

**Figure 1:**
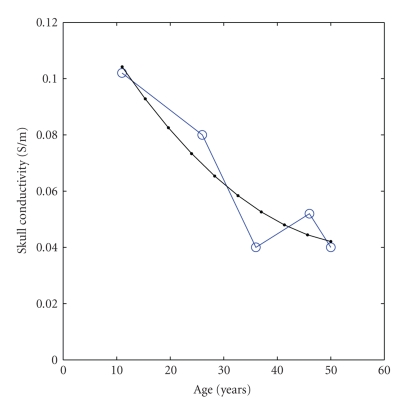
Reported conductivity values of live skull samples temporarily removed during epileptic surgery plotted against patient age [5]. The thick blue trend with circles graphs raw data and the thin gray trend with dots graphs the least squares fit. Reproduced from [6].

Previously, we correlated skull conductivity with age (Figure 1) [6]. In that former study, we analyzed the reported skull conductivities of living skull fragments temporarily excised during epilepsy surgery with the age of the patient [5]. We reported a decreasing trend that stabilized in early adulthood. According to medical texts, physiologists explain that the calvarial bone completes the ossification process between the ages of 18 and 20 [7]; therefore, the skull conductivity should nearly approach steady state after adolescence. From the study of Hoekema et al. [5], we extrapolate that the scalp-to-skull conductivity ratio of 5 represents children and a small percentage of adolescents, the ratio of 8 represents adolescents and some adults, the ratio of 15 represents most adults, and lastly the ratio of 30 represents cadavers suffering from postcellular death. Ages that overlap scalp-to-skull conductivity ratios accommodate inter- and intrasubject variability [1, 8]. Taking standard skin conductivity values [9, 10] divided by the adult skull conductivity values yields a ratio of 8.5, and then scaled by the living to postmortem factor [6, 11, 12] yields approximately 20 to 26. These ratios fit accordingly with [13], which reported a ratio of 15 for post mortem skulls beyond cellular death.

In the present study we apply the concepts of the half-sensitivity volume (HSV) [14] and region of interest sensitivity ratio (ROISR) [15]. We use these metrics to analyze the effects of EEG electrode implantation on the measurement sensitivity distribution within the brain. Specifically, we aim to compare the sensitivity distributions of the bipolar subdermal EEG measurement with the well-documented surface electrode according to a patient's age [6, 14, 16–19]. 

## 2. Methods

### 2.1. Sensitivity Distribution

The sensitivity distributions of measurement leads in an inhomogeneous volume conductor can be illustrated with lead current fields as defined by [20–22]. The lead vectors define the relationship between the measured signal in the lead and the current sources in the volume conductor such that

(1) $$\begin{array}{c} V_{\text{LE}}\left(x\right)=\int_{v}\frac{1}{\sigma }J_{\text{LE}}\cdot J^{i}dv, \end{array}$$

where *V*_LE_(**x**) is the voltage, for example, measured EEG voltage, in the volume conductor *v*. The reciprocal current field **J**_LE_ is the lead field, **J** ^*i*^ (A/cm^2^) is the impressed current density vector in the volume conductor, and *σ* is the conductivity (S/m) [17].

The sensitivity distribution in the volume conductor can be established by applying the reciprocity theorem of Helmholtz with Poisson' equation (2) applied to describe quasistatic bioelectric source-field problems [23, 24]. A source distribution, **J** ^*i*^, containing only reciprocal source currents at the measurement electrodes raises a gradient potential distribution, ∇Φ, that is, measurement sensitivity, according to the linear Poisson equation

(2) $$\begin{array}{c} \nabla \cdot \left(\sigma \nabla \Phi \right)=\nabla \cdot J^{i}\;\left(\text{in}\;\;\Omega \right), \end{array}$$

setting the Neumann boundary conditions equal to zero on the scalp

(3) $$\begin{array}{c} \sigma \left(\nabla \Phi \right)\cdot n=0\;\;\left(\text{on}\;\;\Gamma_{\Omega }\right), \end{array}$$

where **σ** is the electrical conductivity tensor, Φ is the electrical potential, **J** ^*i*^ is the current source density, **n** is a vector normal to the surface, Ω is the volume of the head, and Γ_Ω_ is the surface of the head [25]. 

### 2.2. The Half-Sensitivity Volume

In Malmivuo et al. [14], the concept of the half-sensitivity volume (HSV) was applied to define the volume in which the sensitivity of the measurement lead is concentrated. The HSV is the size of the volume within the source region of the volume conductor, where the magnitude of the sensitivity is at least half of its maximum value. The size of the HSV reflects how focused the region is from which the lead measures bioelectric activity, that is, smaller volumes have a higher measurement resolution and, conversely, larger volumes have a lower measurement resolution. The half-sensitivity volume is thus applied to evaluate the ability of the lead to concentrate the measurement sensitivity.

### 2.3. The Region of Interest Sensitivity Ratio

Väisänen et al. [15] introduced the concept of the region of interest sensitivity ratio (ROISR), which provides a parameter to analyze the specificity of a measurement system. Equation (4) defines ROISR as a ratio between the average sensitivity of a predefined region-of-interest (ROI) volume *v*_ROI_ (5) and the average sensitivity in the rest of the source volume, hereafter called a nonROI volume. The ratio is formulated such that

(4) $$\begin{array}{c} ROISR=\frac{\left(1/\left|v_{\text{ROI}}\right|\right)\int_{v_{\text{ROI}}}\nabla \Phi_{\text{LE}}\left(y,x\right)dy}{\left(1/\left|v_{\text{nonROI}}\right|\right)\int_{v_{\text{nonROI}}}\nabla \Phi_{\text{LE}}\left(y,x\right)dy}, \end{array}$$

where *v*_ROI_ is the ROI source volume (cm^3^) and *v*_nonROI_ is the nonROI source volume (cm^3^).

**Figure 2:**
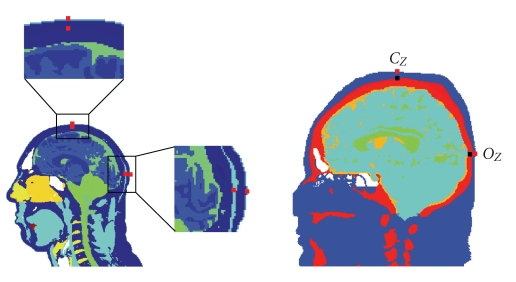
The midsagittal views show the bipolar electrode locations of the surface and subdermal (i.e., on the skull) measurement locations at the apex *C*_*Z*_ and the occipital cortex *O*_*Z*_. The EEG electrode dimensions are 1 mm × 1 mm × 1 mm. (a) The sagittal slice of the *Visible Human Man* displays all four locations. (b) The sagittal slice of the *Visible Human Woman* also shows the surface and subdermal locations.

In the case of EEG, the nonROI volume consists of the entire brain source volume excluding the ROI volume. ROISR thus defines how well the measurement sensitivity is concentrated within the selected ROI, that is, how specific the measurement is to the signals generated within the ROI. We define the ROI volume as

(5) $$\begin{array}{c} v_{\text{ROI}}=v_{B}⋂v_{S}, \end{array}$$

where *v*_*B*_ is the brain source volume containing the gray and white matters, and *v*_*S*_ is a sphere with a 20 mm radius from the cortical electrode located on the occipital cortex surface (10/20 location, *O*_*Z*_, Figure 2). Consequently, our ROI contains both gray and white matters. We selected this location due to its relevance in visually evoked studies by Sörnmo and Laguna [26]. 

### 2.4. Model and Computations

We calculate the sensitivity distributions in a realistically shaped male and female heads model based on the U.S. National Library of Medicine's Visible Human Project digital male and female anatomical dataset [27–29], VHP. Calculation of the sensitivity distributions is based on the principle of reciprocity and the numerical finite difference method (FDM) solution of EEG electrode sensitivity. In the FDM model, the segmented head data from a magnetic resonance image (MRI) data set is divided into cubic elements forming a resistive network [30]. The conductivities, of the elements correspond to the tissue conductivities and the dimensions of the elements correspond to the resolution of the dataset. The FDM is based on Poisson's equation that can be used to describe the bioelectric quasistatic source field problems [24]. A potential distribution within the model for a specific source configuration is solved with linear equations and iterative methods [31, 32].

EEG source localization and head model simulations significantly depend on the conductivities used in the models. In literature many studies apply a brain-to-skull conductivity ratio between 15 and 80 [33]; however, these two parameters vary widely in their conductivity values. The brain tissue conductivity value ranges from 0.12 S/m to 0.48 S/m [1, 8, 34–40], whereas the skull conductivity value ranges from 0.0042 S/m to 0.3 S/m [5, 8, 11, 13, 34–36, 41]. The scalp (skin) conductivity value varies less in literature from 0.33 S/m to 0.45 S/m [8, 9, 34, 35, 42]. Therefore, in the present study we apply the scalp-to-skull conductivity ratios of 5, 8, 15, and 30 : 1 [1, 6, 13, 38–40, 43]. The tissues and their corresponding conductivity values that we used in this study are listed in Table 1 [10].

We calculate the sensitivity distributions of the brain for each bipolar electrode pair located on the scalp and the skull. The surface electrodes (a.k.a. scalp electrodes) and the subdermal electrodes measure 1 mm × 1 mm × 1 mm, which reflects the size of one pixel. These dimensions represent one type of subdermal recording electrodes that are insulated up to the tip. Our bipolar leads reflect a visually evoked measurement over the occipital cortex (10/20 location *O*_*Z*_) referenced against an apex electrode (10/20 location *C*_*Z*_). The sagittal views of the models (Figure 2) show the two bipolar EEG locations: surface electrode on the scalp and the subdermal electrode on the skull.

## 3. Results

**Figure 3:**
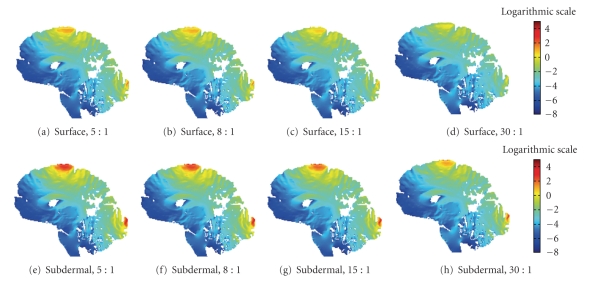
Measurement sensitivity distributions of the *Visible Human Man* mapped in the logarithmic scale: ((a)–(d)) surface electrodes placed on the scalp solved according to the scalp-to-skull conductivity ratio mentioned in the subcaption and ((e)–(h)) subdermal insulated needle electrodes inserted through the skin placing the measuring tip on the skull surface solved according to the scalp-to-skull conductivity ratio mentioned in the subcaption. Scalp-to-skull conductivity ratios are specified in each subcaption: ((a), (e)) 5 : 1, ((b), (f)) 8 : 1, ((c), (g)) 15 : 1, and ((d), (h)) 30 : 1.

**Figure 4:**
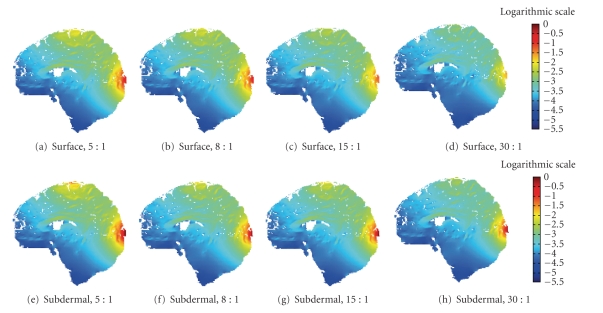
Measurement sensitivity distributions of the *Visible Human Woman* mapped in the logarithmic scale: (a)–(d) surface electrodes placed on the scalp solved according to the scalp-to-skull conductivity ratio mentioned in the subcaption and (e)–(h) subdermal insulated needle electrodes inserted through the skin placing the measuring tip on the skull surface solved according to the scalp-to-skull conductivity ratio mentioned in the subcaption. Scalp-to-skull conductivity ratios are specified in each subcaption: (a), (e) 5 : 1, (b), (f) 8 : 1, (c), (g) 15 : 1, and (d), (h) 30 : 1.

 Figures 3 and 4 present the sensitivity distributions of both the scalp and subdermal leads solved with different conductivity ratios. Clearly, the conductivity ratio has a significant impact on the sensitivity distribution when we consider only one type of electrodes. However, the comparison of both types of electrodes diminishes the influence of the conductivity correlated with age, thus indicating the improved measurement resolution of the needle electrodes irrespective of the patient's age.

Optimally placed subdermal electrodes nearly outperform surface electrodes at every age. The smearing effect of the scalp disappears with the subdermal leads because the recording locations are closer to the target region, thus bypassing the skin (Figures 3 and 4). Tables 2 and 3 show that the subdermal lead's HSV decreases to nearly one-seventh, one-nineth, one-eighth, and one-fourth the size of the scalp lead's HSV. Similarly, we find a 35% to 37% improvement in the subdermal lead's ROISR over the surface lead's ROISR. Figures 3 and 4 illustrate that the subdermal measurement distributions visibly concentrate the measurement sensitivity more efficiently to the target region on the cortex of the younger patient's skull (i.e., lower conductivity values). Moreover, the smearing effect of the skull is reduced with the subdermal leads, and nearly the entire scalp and skull smearing is eliminated when the patient is the youngest (i.e., the skull conducting value is at its peak). Conversely, the older the patient, namely, the higher the scalp-to-skull conductivity ratio, the more the skull conductivity smears the lead field formation. Precisely, the subdermal leads measure neuroelectric activity on or near the gyral cortical surface rather than sulcal or deep sources.

## 4. Discussion

 The present study compares two variables influencing EEG source localization studies: age and electrode location. This study shows that the ratio between the scalp and subdermal measurements regarding the HSV is smallest with the lowest skull conductivity ratio. The correlation between the HSV ratios indicates that measurements will be more localized, that is, increased sensitivity, with higher specificity (ROISR). The subdermal measurement distributions visibly concentrate the measurement sensitivity more efficiently to the target region on the cortex as the skull conductivity increases. The smearing effect of the scalp is reduced with the subdermal leads, and nearly the entire scalp and skull smearing is eliminated when the skull has its highest conducting value [8]. Precisely, the subdermal leads measure neuroelectric activity on or near the gyral cortical surface rather than sulcal or deep sources.

Tissue conductivities such as skin, cortical bone, and brain conductivities change with age [41, 44–50]. Their results indicate a decrease in conductivity between 40.7% and 75.4% from newborn to maturity stages. Furthermore, their results show that the aging process slows during childhood before adolescence after the rapid growth phases of the body have been completed. This is due to the reduction of water content in tissue as a function of age [47, 50]. We believe that the conductivity of the skin changes again in late adulthood, that is, the elderly, particularly decreasing in conductivity. Therefore, the skin conductivity from adolescence onwards should minimally affect this study.

We selected our scalp-to-skull conductivity ratios to span from early childhood through adulthood. Our skull values reflect an 83.9% decrease in the human skull conductivity value compared with the 75.4% change in rats, whereas we kept a fixed conductivity for the brain and skin. When we compare similar sets of measurements such as the surface measurements we obtain an improvement in measurement, resolution between 10.4% and 51.1% for the HSV and an improvement in the measurement accuracy between 25.5% and 38.2% for the ROISR. When we include the subdermal needle measurements, we yield improvements between 75% and 89% in the measurement resolution over the surface electrodes. If we had factored in growth from youth through adolescence to adulthood, then the change in HSV and ROISR would have increased the variation in the results. The additional variables would have plausibly enhanced the measurement precision in childern due to the high water content of their tissues [47].

## 5. Conclusion

 The implantation of EEG electrode on the skull notably increases the measurement sensitivity and accuracy over traditional surface electrodes. These measurements known as subdermal or subcutaneous measurements bypass the artifact prone skin to obtain relatively artifact-free, high-resolution EEG recordings. The measurement sensitivity of the needle electrodes concentrates the subdermal EEG measurements. Consequently, the subdermal electrode reduces the need for the extremely invasive electrocorticogram (ECoG) and minimizes the influence of age on EEG source localization. We found that the scalp-to-skull conductivity ratio influenced the subdermal EEG measurement less than the surface EEG measurements. From our correlative study we can definitively claim that children, specifically preadolescent children, would benefit the most from the increased resolution of the subdermal electrodes.

The age plays an important role in the surface electrode measurements, but the change in measurement location to subdermal electrodes irrefutably improves the measurement sensitivity distributions. Succinctly, the subdermal electrodes outperform surface electrodes because they minimize the effect due to the intersubject variability in the scalp-to-skull conductivity ratio associated with the change in age.

## Figures and Tables

**Table 1:** Tissues and conductivities (S/m) included in our realistic head models [10].

Tissue	Conductivity (S/m)	Tissue	Conductivity (S/m)
Bone marrow	0.046	Scalp	0.43
Fat	0.040	Eye	0.51
Skull/Bones	0.087, 0.054, 0.029, 0.014	Muscles	0.11
White matter	0.14	Blood	1.0
Gray matter	0.33	CSF	1.54
Other neural tissue	0.16		

**Table 2:** Results of the visually evoked bipolar measurement for the surface and subdermal leads of the *Visible Human Man* dataset. All parameters are calculated from the brain region containing both the gray and white matters.

Leads	Conductivity Ratio	Maximum Sensitivity (A/cm^3^)	HSV (mm^3^)	ROISR
Surface	5 : 1	0.420	4999	2.43
Surface	8 : 1	0.405	5239	2.31
Surface	15 : 1	0.387	4002	2.09
Surface	30 : 1	0.336	2446	1.81

Subdermal	5 : 1	0.85	706	3.17
Subdermal	8 : 1	0.83	586	3.00
Subdermal	15 : 1	0.72	516	2.72
Subdermal	30 : 1	0.54	610	2.39

**Table 3:** Results of the visually evoked bipolar measurement for the surface and subdermal leads of the *Visible Human Woman* dataset. All parameters are calculated from the brain region containing both the gray and white matters.

Leads	Conductivity Ratio	Maximum Sensitivity (A/cm^3^)	HSV (mm^3^)	ROISR
Surface	5 : 1	0.900	221	5.95
Surface	8 : 1	0.775	199	5.41
Surface	15 : 1	0.515	190	4.58
Surface	30 : 1	0.379	198	3.68

Subdermal	5 : 1	1.689	44	6.88
Subdermal	8 : 1	1.671	36	6.60
Subdermal	15 : 1	1.595	27	6.20
Subdermal	30 : 1	1.491	25	5.92
